# Heterologous Expression of *SoMYB1* Derived from *Syringa oblata* Enhances Cyanidin Biosynthesis in Tobacco

**DOI:** 10.3390/plants15162492

**Published:** 2026-08-17

**Authors:** Yinglong Yang, Xinyi Du, Pan Yang, Bin Wang, Guangji Ye, Huijun Li, Zhenzhen Zheng

**Affiliations:** 1 Qinghai University, Xining 810016, China; 15500684758@163.com (Y.Y.);; 2 Academy of Agriculture and Forestry Sciences, Qinghai University, Xining 810016, China; 3 Qinghai Plateau Key Laboratory of Tree Genetics and Breeding, Xining 810016, China; 4 Qinghai University Key Laboratory of Tibet Plateau Biotechnology of the Ministry of Education, Xining 810016, China; 5 Laboratory for Research and Utilization of Tibet Plateau Germplasm Resources, Xining 810016, China; 6 Zhengzhou Institute of Urban Landscape Science, Zhengzhou 450051, China

**Keywords:** *Syringa oblata*, *SoMYB1*, genetic transformation, ectopic expression, cyanidin

## Abstract

*Syringa oblata* is a renowned flowering shrub, yet the functional studies on the regulatory factors governing its flower color formation remain limited. In this study, a new MYB transcription factor, named *SoMYB1*, was isolated from the petals of *Syringa oblata* by the homologous cloning technique. The open reading frames of *SoMYB1* was 705 bp in length. Amino acid sequence analysis showed that *SoMYB1* contained the highly conserved R2 and R3 MYB domains. A quantitative real-time PCR analysis revealed that *SoMYB1* was expressed tissue specifically in flowers. Ectopic expression of *SoMYB1* induced anthocyanins accumulation in both vegetative and reproductive tissues of transgenic tobacco lines. Overexpression of *SoMYB1* in tobacco enhanced the expression of *NtAN2* (MYB) and *NtAN1b* (bHLH), and the expression of structural genes *NtCHS*, *NtCHI*, *NtF3H*, *NtF3′H*, *NtDFR*, *NtANS*, *NtUFGT*, and *Nt3RT* increased remarkably. The UPLC-MS/MS analysis of transgenic tobacco leaves showed that the heterologous expression of *SoMYB1* significantly promoted the accumulation of anthocyanin metabolites, especially cyanidin, which accounted for more than 50% of the total anthocyanins in the transgenic tobacco lines. This study elucidates the molecular mechanism by which *SoMYB1* positively regulates anthocyanin biosynthesis in *S. oblata*, providing theoretical and technical support for developing plant resources rich in cyanidin.

## 1. Introduction

Anthocyanins are a class of polyphenolic flavonoids widely distributed in plants. These pigments not only impart vibrant colors to various plant tissues but also possess important medicinal properties, including antioxidant, anti-inflammatory, lipid-lowering, anti-aging, and retinoprotective activities [1,2,3,4,5]. Cyanidin and its glycosides are the most prevalent anthocyanins in plants [6,7]. For instance, cyanidin-3-*O*-glucoside (C3G) and cyanidin-3-*O*-rutinoside (C3R), which are abundant in blackberry, mulberry, black raspberry, sweet cherry, and blackcurrant, exhibit a wide range of biological activities, such as antioxidant, anti-inflammatory, and anti-carcinogenic effects [8,9,10,11]. Liu et al. demonstrated that C3G ameliorates non-alcoholic fatty liver disease (NAFLD) lesions in mice fed a high-fat diet by activating the AMPK/SIRT1/NF-κB (Adenylate-Metabolizing Protein Kinase/Silencing Information Regulator 1/Nuclear Factor κB) pathway. This activation occurs through the upregulation of AMPK and its upstream regulators, alongside the inhibition of NF-κB-mediated inflammatory responses [12]. Furthermore, C3R, extracted from the black raspberry cultivar ‘Jewel’, induces apoptosis in human leukemia HL-60 cells in a dose- and time-dependent manner, without exerting cytotoxic effects on normal human peripheral blood mononuclear cells [13]. Additionally, this compound has shown promise in reducing postprandial glucose levels by inhibiting pancreatic α-amylase and intestinal α-glucosidase [14,15].

The structural complexity of the flavylium cation in anthocyanin poses significant challenges for chemical synthesis, making plant extraction the primary method for obtaining pure monomeric anthocyanins [16]. However, conventional extraction from fruits such as blackberry, blueberry, and mulberry is often complicated and costly due to factors including long growth cycles, seasonal harvest constraints, variable yields, and high labor expenses. In contrast, *Nicotiana tabacum* (Tobacco) serves as an efficient “green bioreactor”. Owing to its high leaf biomass, robust environmental adaptability, and well-established genetic transformation and regeneration systems, tobacco has emerged as a preferred platform for plant-derived recombinant protein production [17]. Recent years have seen tobacco-based systems become a powerful tool for the recombinant production of therapeutic proteins and vaccines. For instance, a groundbreaking study demonstrated the heterologous biosynthesis of baccatin III in tobacco, establishing a synthetic biology platform for the sustainable manufacturing of paclitaxel, a blockbuster anticancer drug [18]. Similarly, during the 2014 Ebola outbreak, Mapp Biopharmaceuticals (USA) and Kentucky BioProcessing (KBP) rapidly developed ZMapp, a tobacco-produced monoclonal antibody (mAb) cocktail targeting Ebola virus glycoproteins, which received FDA emergency use authorization and was administered to patients within eight weeks of development [19]. The platform has since expanded to include anti-β-amyloid mAbs for Alzheimer’s disease and bispecific mAbs targeting colorectal cancer [20]. By integrating the natural growth characteristics of plant cells with advanced genetic engineering, this approach offers an efficient, cost-effective, and safe alternative for bioproduct manufacturing [21].

*Syringa oblata* Lindl., a renowned ornamental shrub in the Oleaceae family, is native to northern China and has a cultivation history spanning over 1000 years [22]. In Tibetan medicine, the stems of *S. oblata* are primarily used to alleviate symptoms such as headaches, forgetfulness, insomnia, and irritability [23]. Additionally, rutin in the leaves of *S. oblata* is the main bioactive compound inhibiting Streptococcus suis biofilm formation [24]. Despite its significant ornamental and economic value, the genetic and breeding research for *S. oblata* remains underdeveloped compared to other flowering plants.

R2R3-MYB transcription factors play pivotal roles in regulating anthocyanin biosynthesis across a wide range of plant species. This regulatory mechanism has been extensively characterized in model plants such as *Arabidopsis thaliana* [25,26], *Nicotiana tabacum* [27], and *Antirrhinum majus* [28], as well as in major crop species, including *Zea mays* [29], *Triticum aestivum* [30], and *Solanum tuberosum* [31]. Similar MYB genes have also been identified in numerous other species, such as *Ipomoea batatas* [32], *Capsicum annuum* [33], *Solanum lycopersicum* [34,35], *Epimedium sagittatum* [36], *Petunia hybrida* [37], *Gerbera hybrida* [38], *Fragaria* × *ananassa* [39], *Malus domestica* [40], *Vitis vinifera* [41,42], *Lycium ruthenicum* [43], and *Camellia sinensis* [44]. Consequently, the identification and functional characterization of R2R3-MYB transcription factors regulating anthocyanin biosynthesis constitute an essential prerequisite for elucidating and genetically modifying floral pigmentation patterns in *S. oblata*.

Current research on the molecular mechanisms of anthocyanin biosynthesis in *S. oblata* has primarily focused on floral tissues. In *S. oblata* petals, anthocyanin biosynthesis is driven by the coordinated action of the phenylpropanoid and anthocyanin pathways [45,46]. Although previous studies indicated that MBW (MYB-bHLH-WD40) complex-dependent regulators participate in anthocyanin biosynthesis throughout the flower opening process [45], the specific functions of individual R2R3-MYB transcription factors remain largely unverified. Furthermore, while the general roles of R2R3-MYB factors have been extensively validated through overexpression in model species like tobacco, few studies have specifically examined changes in anthocyanin metabolite profiles in this context. Therefore, this study aims to clone and characterize an R2R3-MYB transcription factor from lilac and conduct targeted metabolome profiling in transgenic tobacco lines. These efforts will provide theoretical insights and genetic resources for elucidating the molecular mechanisms underlying lilac flower coloration and identifying R2R3-MYB transcription factors with specific regulatory functions.

## 2. Results

### 2.1. Sequence and Structural Analysis of SoMYB1

Using *S. oblata* genome data [22] as a reference, we cloned the *SoMYB1* coding sequence (CDS) via combined cDNA library screening and PCR amplification. The open reading frame (ORF) of *SoMYB1* (GenBank accession no. PX516691.1) was 705 bp in length, encoding 234 amino acids (Figures S1–S3), with a calculated molecular weight of 26.83 kDa and an isoelectric point of 9.16.

### 2.2. Phylogenetic Analysis of SoMYB1

To elucidate the evolutionary relationships between *SoMYB1* and other anthocyanin-regulating MYB transcription factors, we performed phylogenetic analysis using amino acid sequences obtained from the NCBI database.

**Figure 1:**
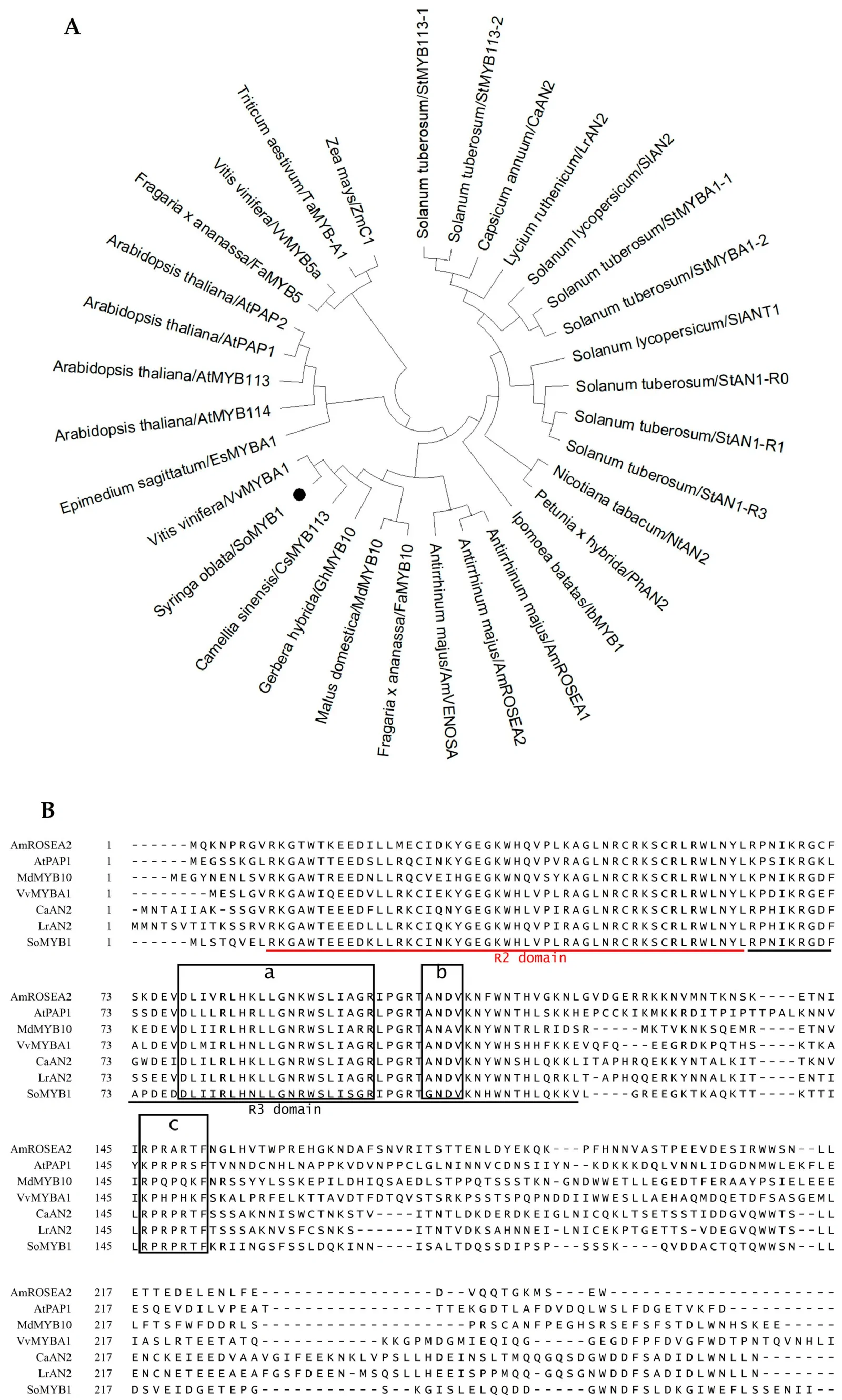
(**A**) Phylogenetic relationship between SoMYB1 and known anthocyanin-related MYB transcription factors in other species. (**B**) The alignment of the amino acid sequences of SoMYB1 and anthocyanin-related MYB transcription factors in other species. The R2 and R3 domains are outlined in red and black frames, respectively. Box (a) indicates the conserved region of the bHLH-interacting motif ([DE]Lx2[RK]x3Lx6Lx3R). Box (b) indicates a conserved motif [A/S/G]NDV in the R2R3 domain for dicot anthocyanin-promoting MYBs. Box (c) indicates a conserved motif [R/K] Px[P/A/R]xx[F/Y] for anthocyanin-regulating MYBs. The accession number of these proteins follows the GenBank database: *Syringa oblata*/SoMYB1:YCO72734.1; *Vitis vinifera*/VvMYBA1: BAD18977.1; *Camellia sinensis*/CsMYB113: UWI70498.1; *Gerbera hybrida*/GhMYB10: CAD87010.1; *Malus domestica*/MdMYB10: ABB84753.1; *Fragaria × ananassa*/FaMYB10: USN17647.1; *Antirrhinum majus*/AmVENOSA: ABB83828.1; *Antirrhinum majus*/AmROSEA2: ABB83827.1; *Antirrhinum majus*/AmROSEA1: ABB83826.1; *Ipomoea batatas*/IbMYB1: BAF45114.1; *Petunia × hybrida*/PhAN2: AAF66727.1; *Nicotiana tabacum*/NtAN2: ACO52470.1; *Solanum tuberosum*/StAN1-R3: AKA95393.1; *Solanum tuberosum*/StAN1-R1: AKA95392.1; *Solanum tuberosum*/StAN1-R0: AKA95391.1; *Solanum lycopersicum*/SlANT1: AAQ55181.1; *Solanum tuberosum*/StMYBA1-2: ALA13582.1; *Solanum tuberosum*/StMYBA1-1: ALA13581.1; *Solanum lycopersicum*/SlAN2: ACT36604.1; *Lycium ruthenicum*/LrAN2: QCS14090.1; *Capsicum annuum*/CaAN2: CAE75745.1; *Solanum tuberosum*/StMYB113-2: ALA13584.1; *Solanum tuberosum*/StMYB113-1: ALA13583.1; *Zea mays*/ZmC1: AAA33482.1; *Triticum aestivum*/TaMYB-A1: AQM56964.1; *Vitis vinifera*/VvMYB5a: AAS68190.1; *Fragaria × ananassa*/FaMYB5: USN17642.1; *Arabidopsis thaliana*/AtPAP2: AAG42002.1; *Arabidopsis thaliana*/AtPAP1: AAG42001.1; *Arabidopsis thaliana*/AtMYB113: OAP11934.1; *Arabidopsis thaliana*/AtMYB114: NP_176812.1; *Epimedium sagittatum*/EsMYBA1: AGT39060.1.

The phylogenetic analysis revealed that *SoMYB1* clustered most closely with VvMYBA1 (*Vitis vinifera*), CsMYB113 (*Camellia sinensis*), and GhMYB10 (*Gerbera hybrida*) (Figure 1A). Multiple sequence alignment of *SoMYB1* with characterized anthocyanin-regulating MYB transcription factors from diverse species—including AmROSEA2 (*Antirrhinum majus*), AtPAP1 (*Arabidopsis thaliana*), MdMYB10 (*Malus domestica*), VvMYBA1 (*Vitis vinifera*), CaAN2 (*Capsicum annuum*), and LrAN2 (*Lycium ruthenicum*)—revealed the presence of conserved R2 and R3 MYB domains in the N-terminal region of *SoMYB1*. This confirms its classification as a functional R2R3-MYB regulator (Figure 1B). Furthermore, *SoMYB1* contains several functionally significant conserved motifs: (1) the ([DE]Lx2[RK]x3Lx6Lx3R) motif (Box a, Figure 1B), which mediates interaction with bHLH cofactors [47]; (2) the GNDV motif (Box b, Figure 1B), previously characterized in anthocyanin-regulating MYBs of the Rosaceae family [48]; and (3) the [R/K]Px[P/A/R]xx[F/Y] motif (Box c, Figure 1B), which is highly conserved among anthocyanin-activating MYB transcription factors across diverse plant species [48].

### 2.3. Expression Patterns of SoMYB1

**Figure 2:**
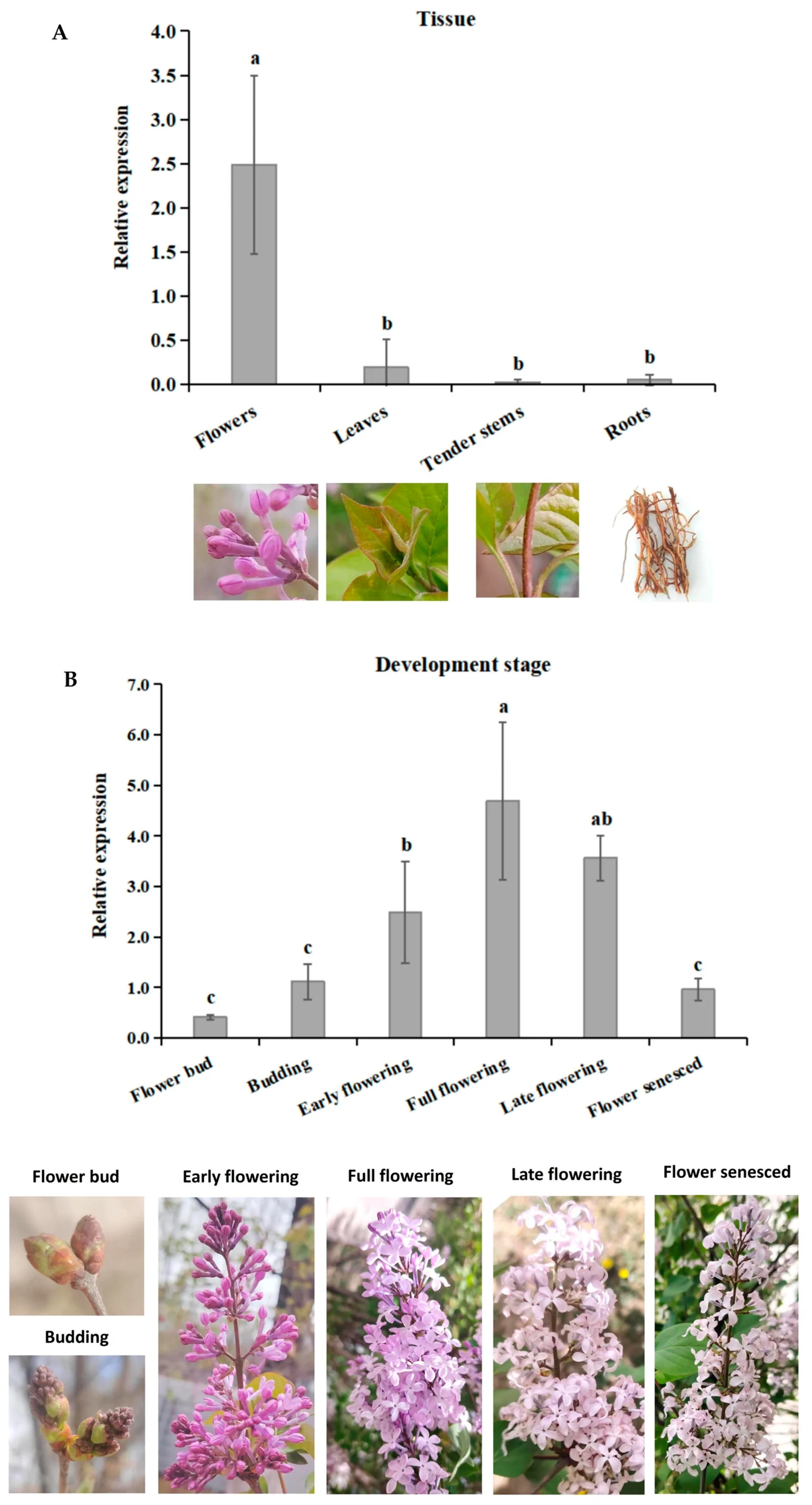
(**A**) *SoMYB1* expression levels of *S. oblata* in the different tissues. (**B**) *SoMYB1* expression levels of *S. oblata* flowers at different developmental stages. Flower bud: flower bud stage with flower buds enlarged and bud scales not protruded; budding: budding stage; early flowering: early flowering stage with a few flowers open; full flowering: flowering stage with flowers fully open and emitting fragrance; late flowering: late flowering stage with some flowers senesced; flower senesced: flowers senesced stage. Data are expressed as means ± standard deviations (SDs) of at least three biological and technical replicates. Differences among tissues were considered significant at *p* < 0.05, as determined by one-way ANOVA.

Real-time qPCR was used to analyze the transcript expression levels of *SoMYB1* in different tissues (flowers, leaves, tender stems, and roots) and at various floral developmental stages of *S. oblata* (Figure 2).

*SoMYB1* transcripts were detected in all examined tissues of *S. oblata*, with significantly higher expression in flowers than in leaves, tender stems, and roots (Figure 2A). Furthermore, *SoMYB1* transcript levels exhibited dynamic changes during floral development, gradually increasing during early flowering, peaking at the full flowering stage, and subsequently declining during late flowering and senescence (Figure 2B).

### 2.4. Overexpression of SoMYB1 Induces Anthocyanin Biosynthesis in Tobacco

**Figure 3:**
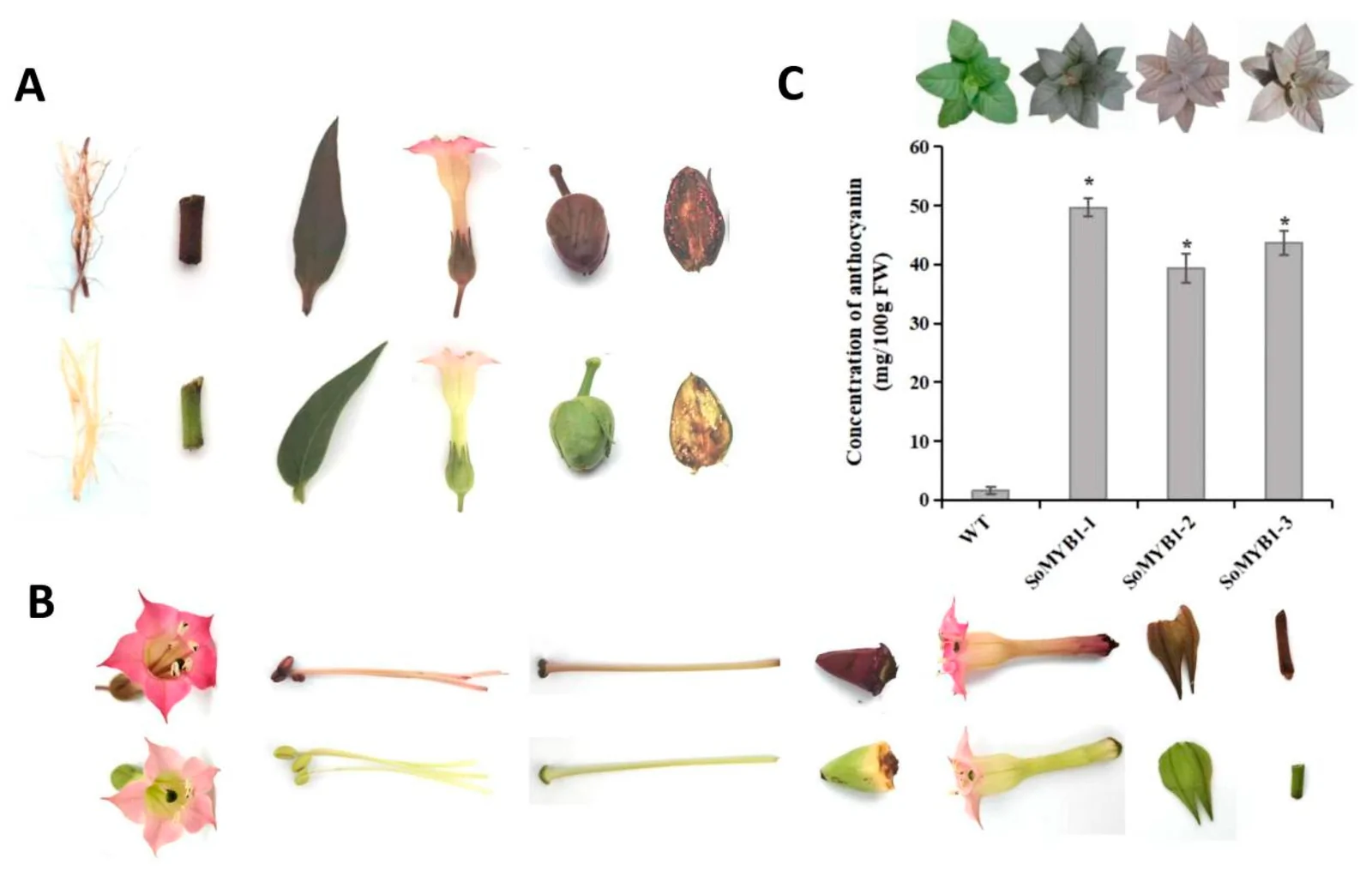
Phenotype and anthocyanin content of transgenic tobacco lines. (**A**) The phenotype of roots, stems, leaves, flowers, capsules, and seeds in transgenic lines (**upper**) and WT (**lower**). (**B**) Phenotypes of the corolla, stamen, stigma and style, ovary, corolla tube, sepal, and peduncle of flowers in the transgenic lines (**upper**) and WT (**lower**). (**C**) The anthocyanin content in the leaves of transgenic lines and WT. Data are expressed as means ± SD of at least three biological replicates. Differences between the transgenic tobacco lines (*SoMYB1*-1, *SoMYB1*-2, *SoMYB1*-3) and WT were considered significant at *p* < 0.05, as determined by Student’s *t*-test. *: Significant differences (*p* < 0.05).

*SoMYB1* was transformed into tobacco plants via Agrobacterium-mediated transformation, yielding seven independent transgenic lines (Figure S5). Phenotypic analysis revealed that ectopic expression of *SoMYB1* induced anthocyanin accumulation in both vegetative and reproductive tissues. Wild-type (WT) plants exhibited white roots and green stems, leaves, capsules, seeds, as well as pale pink flowers. In contrast, all transgenic lines displayed purple pigmentation across all tissues, including roots, stems, leaves, flowers, capsules, and seeds (Figure 3A). Notably, the flower organs of *SoMYB1*-overexpressing plants exhibited significantly higher anthocyanin accumulation than those of the WT, characterized by intense purple pigmentation in the corollas, stamens, stigma-style complexes, ovaries, corolla tubes, sepals, and peduncles (Figure 3B). The three transgenic lines with the most intense pigmentation were selected for quantitative anthocyanin analysis. Among these, line *SoMYB1*-1 exhibited the highest anthocyanin content (50 ± 1.5 mg 100 g^−1^ FW in leaves), while the other selected lines also showed significantly elevated pigment levels compared to the WT (Figure 3C).

qRT-PCR was used to analyze the expression of genes involved in the anthocyanin biosynthesis pathway in the leaves of the *SoMYB1-*overexpressing tobacco line (*SoMYB1*-1). The analyzed genes included three transcription factors (*SoMYB1*, *NtAN2*, and *NtAN1b*) and nine structural genes (*NtCHS*, *NtCHI*, *NtF3H*, *NtF3′H*, *NtF3′5′H*, *NtDFR*, *NtANS*, *NtUFGT*, and *Nt3RT*). The *SoMYB1* transcript levels were substantially upregulated in *SoMYB1*-1 compared to WT. Furthermore, the transcript levels of the endogenous anthocyanin regulatory factors, namely the MYB transcription factor *NtAN2* and the bHLH transcription factor *NtAN1b*, were also significantly elevated in *SoMYB1*-1.

**Figure 4:**
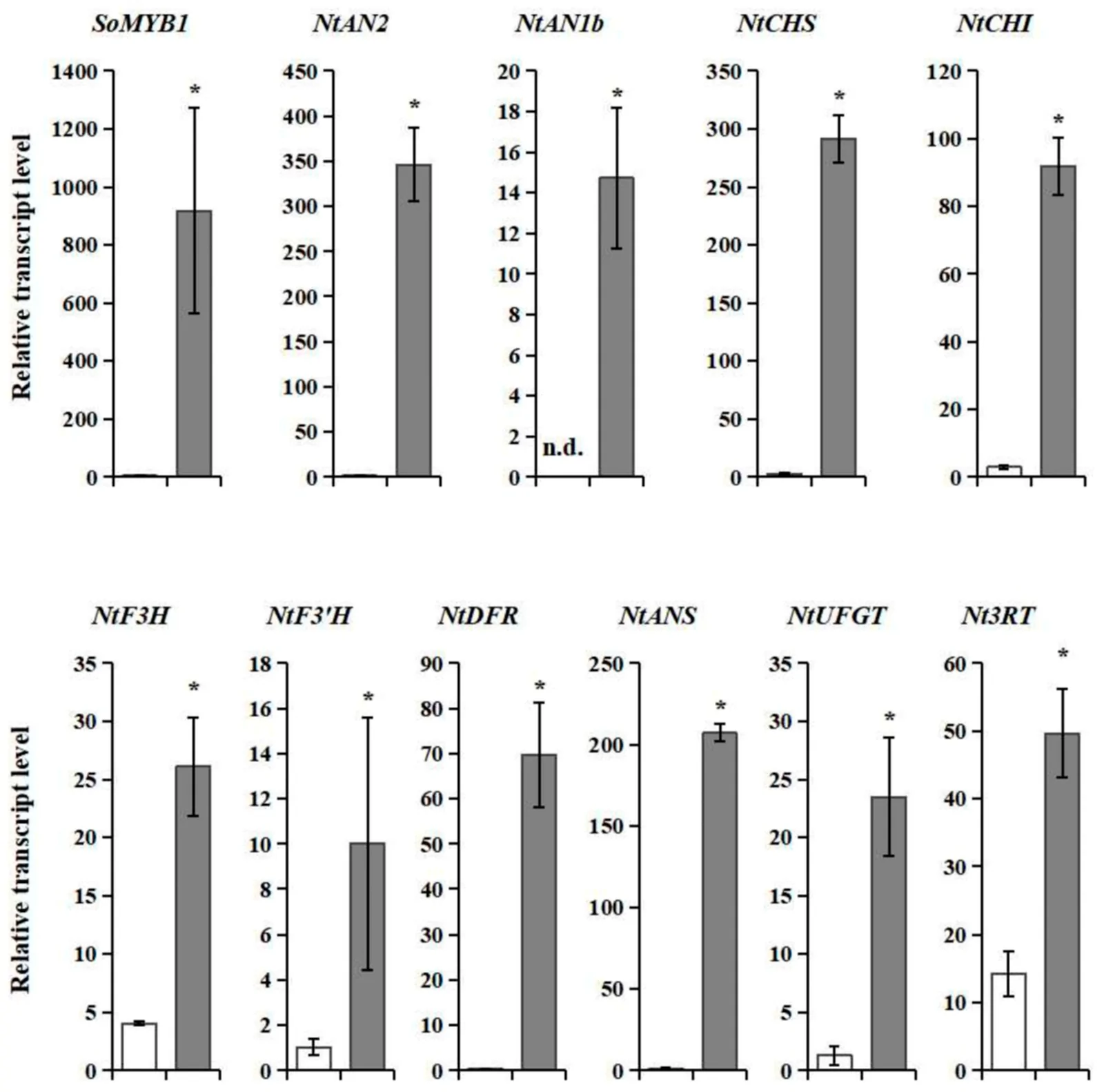
The expression of genes related to the anthocyanin biosynthesis pathway in the leaves of greenhouse-grown wild-type (WT) and transgenic tobacco lines (*SoMYB1*-1). Relative transcript levels in leaves. The white bars represent WT, and the dark grey bars represent *SoMYB1*-1. Data are means ± SD of at least three biological and technical replicates. Differences between *SoMYB1*-1 and WT were considered significant at *p* < 0.05, as determined by Student’s *t*-test. *: Significant differences (*p* < 0.05), n.d.: Not detected.

Among the structural genes analyzed, eight anthocyanin biosynthesis genes—*NtCHS*, *NtCHI*, *NtF3H*, *NtF3′H*, *NtDFR*, *NtANS*, *NtUFGT*, and *Nt3RT*—exhibited significantly higher expression in *SoMYB1*-1 compared to WT, whereas *NtF3′5′H* was not detected (Figure 4).

### 2.5. Chemical Compounds Difference in Transgenic Tobacco Lines and WT

To further characterize anthocyanin metabolites in transgenic and WT tobacco leaves, a targeted metabolomic analysis was performed using a UPLC-ESI-MS/MS system. The total ion flow charts (TICs) of quality control samples demonstrated high repeatability and reliability, confirming the suitability of the data for subsequent analysis (Figure S7).

**Figure 5:**
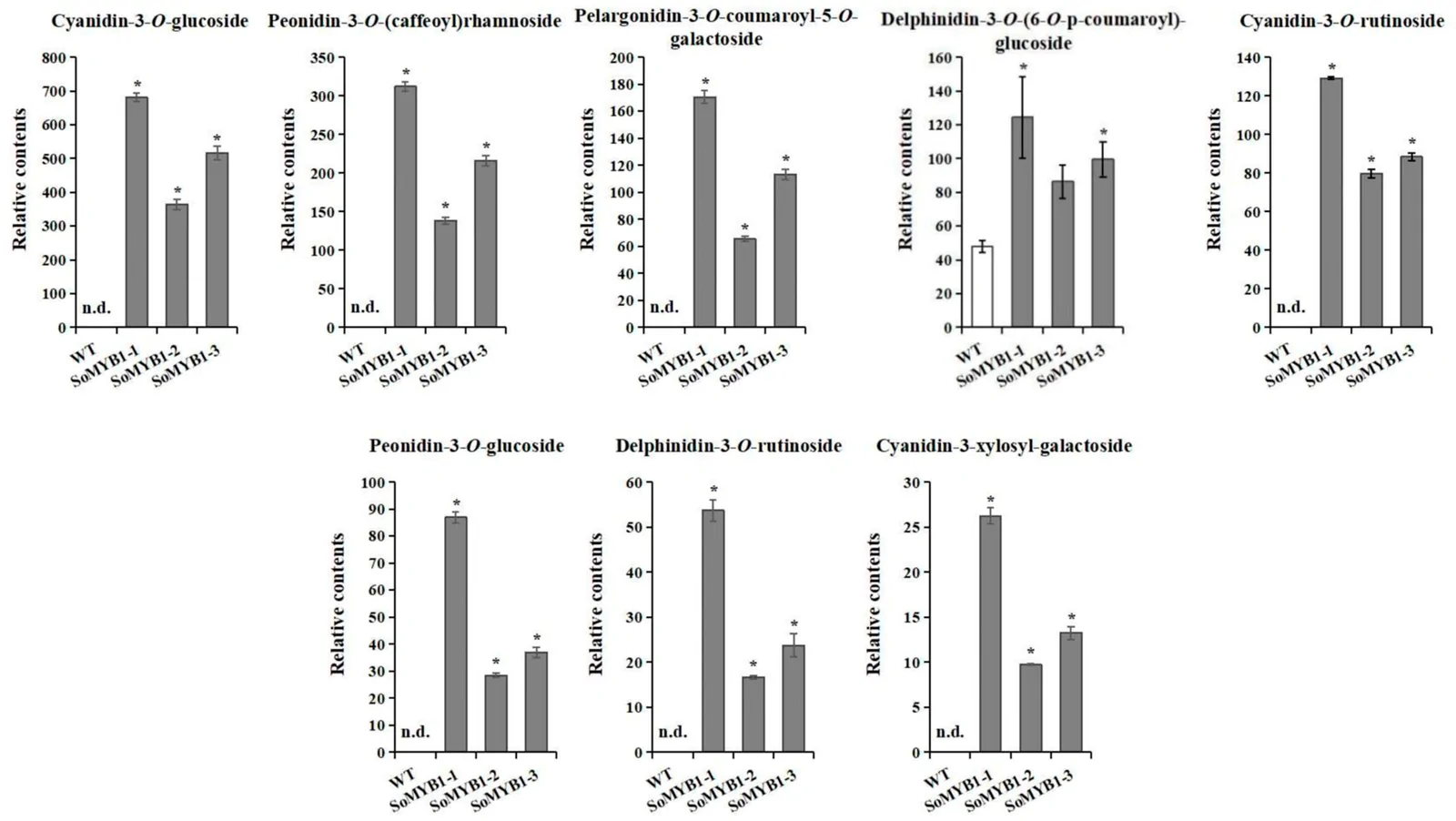
The contents of anthocyanin differential metabolites of the transgenic lines (*SoMYB1*) of T1 and WT in tobacco leaves. Data are expressed as means ± SD of at least three biological replicates. The difference between the transgenic tobacco lines (*SoMYB1*-1, *SoMYB1*-2, and *SoMYB1*-3) and WT was considered significant at *p* < 0.05, as determined by Student’s *t*-test. *: Significant differences (*p* < 0.05), n.d.: Not detected.

A total of 61 chemical compounds were identified across all samples, comprising 57 anthocyanins, two flavonoids, and two procyanidins. Among these, 38 compounds exhibited significantly different accumulation between transgenic lines and WT, including 36 anthocyanins, one flavonoid, and one procyanidin (Table S2). Among the 36 anthocyanins analyzed, 31 exhibited significantly higher levels in transgenic lines compared with WT (Table S2), while five showed decreased accumulation. Notably, only eight of these anthocyanins exceeded a concentration of 10 μg/g dry weight (DW) (Figure 5).

The anthocyanin metabolite class with the highest content comprised cyanidin derivatives (663.88 μg/g DW), accounting for 53.00% of the total anthocyanin content in transgenic leaves. Cyanidin-3-*O*-glucoside was the most abundant cyanidin derivative (520.44 μg/g DW), representing 41.20% of the total anthocyanin content. Cyanidin-3-*O*-rutinoside ranked fifth, with a content of 90.03 μg/g DW.

## 3. Discussion

### 3.1. SoMYB1 Has a Mechanism for Inducing Anthocyanin Synthesis

In plants, anthocyanins are biosynthesized via the phenylpropanoid pathway and transcriptionally regulated by the MBW protein complex [31]. Among these regulatory components, MYB transcription factors play a pivotal role in controlling the expression of anthocyanin biosynthesis genes in various species, including *Arabidopsis* [26], potato [31], wolfberry [43], grape [42], and apple [40]. Here, we identified a novel R2R3-MYB transcription factor, designated *SoMYB1*, from the ornamental plant *S. oblata*. Phylogenetic analysis indicated that *SoMYB1* shares the highest sequence similarity with VvMYBA1, CsMYB113, and GhMYB10 (Figure 1). Previous studies have shown that transgenic tobacco (*N. tabacum* cv. ‘Samsun’) plants expressing *VvMybA1* driven by various promoters—including the double-enhanced CaMV35S (d35S), double-enhanced CsVMV (dCsVMV), a bi-directional dual promoter (BDDP), or *Vitis vinifera* ubiquitin promoters (VvUb6-1 and VvUb7-2)—accumulate anthocyanin in sepals and corollas [49,50]. Additionally, *GhMYB10* induces anthocyanin pigmentation in transgenic tobacco, particularly in vegetative tissues and anthers [38]. In this study, functional validation via ectopic expression showed that *SoMYB1*, driven by the double-enhanced CaMV35S promoter, was transformed, significantly increased anthocyanin accumulation in various tissues, including vegetative parts, sepals, corollas, and anthers (Figure 3). The phenotypic variations observed in stable transgenic lines likely result from difference in the expression vector, *Agrobacterium tumefaciens* strain, and promoter configurations used for *VvMybA1* [49,50], *GhMYB10* [38], and *SoMYB1* (in this study).

Furthermore, *SoMYB1* exhibits flower-specific expression, with transcript levels gradually increasing during floral development and peaking at the full flowering stage (Figure 2). This expression pattern correlates with the period of flower color transition. Our findings are consistent with those of Ma et al. [45], who showed that the MBW complex plays an essential role in flower color formation in *S. oblata*.

In tobacco, *NtAN2* (an R2R3-MYB) and *NtAN1b* (a bHLH) are important components of the MBW complex that regulate anthocyanin biosynthesis [31,51]. Previous studies showed that overexpressing of *StAN1-R1* in tobacco significantly increased anthocyanin accumulation in both leaves and flowers; however, this resulted in the strong induction of *NtAN1b* expression but not *NtAN2* [31]. In contrast, when heterologous expressed *SoMYB1* in tobacco, the expression of endogenous MBW complex components (*NtAN2* and *NtAN1b*) significantly upregulated. Then the expression of structural genes, including *NtCHS*, *NtCHI*, *NtF3H*, *NtF3′H*, *NtDFR*, *NtANS*, *NtUFGT*, and *Nt3RT* increased (Figure 4). Consequently, anthocyanin accumulation occurred, resulting in the purple pigmentation of the transgenic tobacco plants. NtUFGT (flavonoid 3-O-glucosyltransferase) and Nt3RT (UDP-rhamnose anthocyanidin-3-glucoside rhamnosyltransferase) catalyze the final steps in the biosynthesis of C3G and C3R, respectively [52,53]. Previous studies have shown that the recombinant *Freesia hybrida *enzyme Fh3GT1 (a UDP-glucose: anthocyanidin 3-*O*-glucosyltransferase) catalyzes the conversion of cyanidin to C3G in the presence of UDP-glucose [54]. Similarly, a cDNA clone from *Petunia hybrida*, corresponding to the *Rt* locus, encodes a UDP rhamnose:anthocyanidin-3-*O*-glucoside rhamnosyltransferase (3RT) that converts anthocyanidin-3-*O*-glucosides to anthocyanidin-3-*O*-rutinosides [53]. In this study, significantly elevated expression levels of *NtUFGT* and *Nt3RT* consistently correlated with higher concentrations of C3G and C3R in transgenic tobacco plants. However, the direct interactions between SoMYB1 and NtAN2/NtAN1b, as well as the specific binding mechanisms of these complexes to the promoters of *NtUFGT* and *Nt3RT*, require further validation.

Notably, while *NtF3′H* expression was significantly upregulated in *SoMYB1-*transgenic plants, *NtF3′5′H* expression remained negligible in both transgenic lines and WT. This indicates that *SoMYB1* does not induce *NtF3′5′H*, thereby restricting anthocyanin biosynthesis to the cyanidin pathway. Consequently, the accumulated anthocyanins in transgenic plants consisted predominantly of cyanidin and its derivatives, a finding confirmed by targeted metabolomics. Consistent with our findings, gene expression analysis in *LrAN2*-overexpressing tobacco revealed that *NtF3′H* was the primary differentially expressed gene, while *NtF3′5′H* showed no significant changes [55]. Given this limitation, engineering novel pigmentation patterns by overexpressing *NtF3′5′H* in transgenic plants represents a strategy to expand the color diversity of tobacco and other plants.

### 3.2. SoMYB1 Can Be Used to Create Plants Rich in Cyanidin

In plants, anthocyanins typically exist as complex mixtures of various anthocyanidin-glycosides. The specific composition and concentration of these pigments vary significantly depending on factors such as cultivar, season, climate, and developmental stage. Consequently, isolating individual components for functional analysis requires rigorous purification protocols, including multiple ethanol extractions followed by High-Performance Liquid Chromatography (HPLC) [16].

Recent studies have demonstrated that C3G and C3R ameliorate hepatic damage induced by high-fat diet [11,15]. Previous studies validating heterologous MYB transcription factors in tobacco have typically relied solely on total anthocyanin quantification, often neglecting the specific composition of anthocyanin profiles. In contrast, this study presents a quantitative analysis of individual anthocyanin components in *SoMYB1*-transgenic tobacco plants. In the present study, *SoMYB1*-transgenic lines showed significantly elevated total anthocyanin content compared to WT. Notably, cyanidin accumulation in transgenic lines reached 663.88 μg/g DW, constituting 53.00% of the total anthocyanins, with C3G and C3R accounting for 91.96% of the total cyanidin content. While Zong et al. [55] utilized non-targeted metabolomics to profile anthocyanins in *LrAN2-*transgenic tobacco—reporting the significant accumulation of cyanidin 3,5-*O*-diglucoside, pelargonidin 3-*O*-beta-D-glucoside, and apigeninidin chloride—our targeted quantitative analysis yielded divergent results. These discrepancies indicate likely species-specific regulatory patterns of heterologous MYB transcription factors within the tobacco anthocyanin biosynthesis pathway, suggesting that sequence variations may lead to distinct functional outcomes that require further investigation.

It is also important to note that the expression vector *PJAM1502* used in this study contains a double 35s promoter, which drives transgene expression in all tissues. Consistent with findings from Zong et al. [43], both *LrAN2* and *LbAN2* induced anthocyanin biosynthesis in the roots, stems, leaves, flowers and seeds of tobacco. However, despite the increased anthocyanin content, the relative proportion of anthocyanins to leaf dry weight remains low, necessitating further optimization. Furthermore, while *SoMYB1* overexpression elevates anthocyanin levels, it may impose a metabolic burden that could adversely affect plant growth and stress resistance. Consequently, to achieve higher anthocyanin accumulation specifically in leaves, future studies could employ vectors with tissue-specific promoters.

## 4. Materials and Methods

### 4.1. Plant Materials

All plant materials were maintained at the Academy of Agriculture and Forestry Sciences, Qinghai University, under optimal growing conditions. *Syringa oblata* Lindl. plants were cultivated in the germplasm resources nursery; plants used in this study were over 10 years old. *Nicotiana tabacum* “Samsun” plants used for transformation were maintained in a controlled growth chamber at 25 °C with a 16 h photoperiod and a light intensity of 2000 lx in a tissue culture room. WT and transgenic tobacco plants were subsequently grown in a greenhouse under standard conditions. All samples were flash-frozen in liquid nitrogen and stored at −80 °C until use.

### 4.2. Isolation of SoMYB1 from S. Oblata

To obtain the CDS of *SoMYB1*, we aligned MYB transcription factors known to regulate anthocyanin biosynthesis from *Lycium ruthenicum*, *Solanum tuberosum*, and *Arabidopsis thaliana* using Invitrogen Alignx software10.3.0 Based on the conserved motifs identified, the *SoMYB1* CDS was retrieved from the *S. oblata* genome data [22]. Total RNA was extracted from *S. oblata* flowers using the TaKaRa MiniBEST Universal RNA Extraction Kit (TaKaRa, Beijing, China) according to the manufacturer’s instructions. cDNA was synthesized from the total RNA using the PrimeScript^TM^ 1st Strand cDNA Synthesis Kit (TaKaRa). Initial primer designs based on the predicted CDS failed to yield amplification products from *S. oblata* cDNA templates. Subsequently, we identified the *SoMYB1* DNA sequence and utilized *S. oblata* genomic data to assemble the upstream and downstream sequences, obtaining a 1638 bp *SoMYB1* gene sequence. Based on this assembled sequence, we designed primers (*SoMYB1*-F and *SoMYB1*-R) to amplify *S. oblata* cDNA, yielding a 983 bp sequence that includes the *SoMYB1* CDS region. Next, we recovered the PCR products, ligated the target fragments into the PLB vector, and sent the positive strains to Shanghai Biotech for sequencing, with accurate results obtained. PCR reactions (20.0 µL total volume) were prepared using 1.0 µL cDNA, 0.5 µL each of the forward and reverse primers (10 µM), 10.0 µL of 2 × *Taq* PCR MasterMix II, and 8.0 µL of ddH_2_O. Amplification was carried out using the cycling parameters recommended by the manufacturer. Primers were designed using NCBI’s Primer-BLAST tool (https://www.ncbi.nlm.nih.gov/tools/primer-blast/index.cgi?LINK_LOC=BlastHome) (accessed on 1 June 2023) and synthesized by Sangon Biotech (Shanghai) Co., Ltd(Shanghai, China).

### 4.3. Gene Sequence and Phylogenetic Analysis

The open reading frame (ORF) of *SoMYB1* was translated into an amino acid sequence using NovoPro (https://www.novopro.cn/tools/translate.html) (accessed on 1 October 2023). Protein physicochemical properties, including molecular weight and isoelectric point, were analyzed using the ProtParam tool from Expasy (http://web.expasy.org/) (accessed on 1 May 2024). The protein sequences of the homologous MYB transcription factors known to regulate anthocyanin biosynthesis from other plants, were obtained using NCBI BLAST. Phylogenetic analysis was performed using MEGA11. The protein sequences were aligned using the Align by clustalW, and a phylogenetic tree was constructed using the neighbor-joining method with 1000 bootstrap replicates.

### 4.4. qRT-PCR

Tissues were collected from *S. oblata* and *N. tabacum.* For *S. oblata*, flowers, leaves, stems, and roots at various developmental stages were harvested. For *N. tabacum*, leaves from both WT and transgenic plants grown in the greenhouse were collected. All samples were immediately flash-frozen in liquid nitrogen and stored at −80 °C until RNA extraction. Total RNA was isolated from various tissues using the TaKaRa MiniBEST Universal RNA Extraction Kit (TaKaRa, Beijing, China) according to the manufacturer’s instructions. First-strand cDNA was synthesized from total RNA using the PrimeScript^TM^ RT Master Mix (Perfect Real Time) (TaKaRa). Quantitative real-time PCR (qRT-PCR) was performed using TB Green^®^ Premix Ex Taq^TM^ (Tli RNaseH Plus) (TaKaRa). Each experiment included at least three biological replicates, and each replicate was performed in triplicate (technical replicates). The expression levels of *S. oblata* and *N. tabacum* samples were normalized to the reference genes *SoActin* and *NtELF*, respectively. The average relative expression levels in the transformed lines were calculated using the 2^−ΔΔCt^ method. To distinguish between the homologous genes *NtAN2* and *SoMYB1*, qRT-PCR primers were designed using sequences unique to each gene, thereby ensuring high specificity and avoiding cross-reactivity. The sequences of the qRT-PCR primers are listed in Supplementary Table S1.

### 4.5. Expression Vector Construction

Primers *SoMYB1*-*attB*-F and *SoMYB1*-*attB*-R (Supplemental Table S1) were designed in accordance with the Gateway Cloning kit manual (Invitrogen). The *SoMYB1*-CDS sequence was ligated into the entry vector *pDONR207* to generate the entry clone *pDONR207*-*SoMYB1*, and the *pDONR207*-*SoMYB1* was reconstituted with the expression vector *PJAM1502* to obtain the expression clone *PJAM1502*-*SoMYB1* (Figure S4). The steps of vector construction followed the protocols outlined in the Gateway Cloning Kit manual (Invitrogen). The binary vector *PJAM1502* contains a double 35S promoter [43]. The resulting plasmid was transformed into *Escherichia coli* DH5α competent cells. Positive clones were selected, and plasmids were prepared for subsequent Agrobacterium-mediated transformation.

### 4.6. Transformation of Tobacco

The *PJAM1502*-*SoMYB1* expression vector was transferred into *A. tumefaciens* strain GV3101 by the freeze-thaw method. Leaves obtained from 2- to 3-week-old seedlings were cut into pieces about 1 cm in length and 1 cm in width, incubated for 10 min in a culture (OD600 = 0.6) of *A. tumefaciens* GV3101 harboring *PJAM1502*-*SoMYB1*. The explants were blotted dry and then placed on basal medium and cocultivated for 2 days in the dark. The basal medium as described by Zheng et al. (2021) [56]. This was followed by transferring the explants to a fresh basal medium containing cefotaxime (300 mg/L) and incubated for 3–5 days under illumination 2000 lx at 25 °C. Selection was then initiated by transferring the explants to basal medium containing cefotaxime (300 mg/L) and kanamycin (50 mg/L, Diamond, Shanghai, China). When the green shoots emerging from the explant reached approximately 1.5 cm in length, they were transferred onto sterile culture vessels with MS medium containing cefotaxime (300 mg/L) and kanamycin (50 mg/L) and maintained at 25/20 °C (day/night). The genomic DNA was extracted from wild-type *N. tabacum* and transformant plants using the DNAsecure Plant Kit System (Tiangen). Transgenic plants were preliminarily identified (Figure S5) via PCR amplification using the primers SoMYB1-attB1-F and SoMYB1-attB2-R. Subsequently, qRT-PCR was performed to analyze *SoMYB1* expression levels in the leaves of the T1-generation transgenic lines that exhibited significant anthocyanin accumulation (Figure S6). Since only T1-generation transgenic lines were obtained in this experiment, chimeric individuals were present, and no clear gene segregation pattern was observed.

Primers com-F (5′-CACTCTTTCCCTACACGACGCTCTTCCGATCT-3′) and com-R (5′-GACTGGAGTTCAGACGTGTGCTCTTCCGATCT-3′) were designed targeting the LB-R (5′-GACTGGAGTTCAGACGTGTGCTCTTCCGATCTaaccaccccagtacatta-3′) and RB-F (5′-CACTCTTTCCCTACACGACGCTCTTCCGATCTtgactcccttaattctcc-3′) regions. Uppercase letters represent the reference genome sequence, while lowercase letters represent the vector sequence. A whole-genome resequencing approach was employed to distinguish between different transgenic lines using barcodes. Sequencing data were processed using Fastp (v0.1.2.4) for quality control and the removal of primers and adapters. Subsequently, the BWA (v.0.7.17) MEM program was employed to align the sequencing reads to the reference genome. Analysis was then performed using SAMtools and Perl scripts to obtain information regarding insertion sites; differences in POS values reflect variations between different transgenic lines (Table S3).

### 4.7. Anthocyanin Content of Transgenic Tobacco

The anthocyanin content was quantified in the leaves of WT and transgenic lines following the method described by Jia et al. [57]. The experiment included at least three biological replicates, and each replicate was performed in triplicate (technical replicates). Leaves from WT and transgenic tobacco plants were harvested. Precisely 0.1 g of leaf tissue was weighed and placed into a 50 mL brown volumetric flask. Twenty-five milliliters of extraction solution (conc. HCl:80% methanol, 1:1000, *v*/*v*) was added. The flask was tightly sealed, weighed, and subjected to sonication for 1 h. After sonication, the flask was re-weighed, and any mass loss was compensated for by adding the extraction solution. The mixture was vortexed thoroughly to ensure homogeneity. For each sample, two aliquots were prepared: one diluted with KCl buffer (0.025 M, pH 1.0) and the other with sodium acetate buffer (0.4 M, pH 4.5), both at a ratio of 1:4 (test solution:buffer, *v*/*v*). Absorbance was measured at 530 and 700 nm within 20–50 min after dilution, and the anthocyanin content was calculated based on these values [57]. Calculate anthocyanin pigment concentration, expressed as cyanidin-3-glucoside equivalents, as follows: C (anthocyanin pigment) = A × M × DF × 10^3^/(ε × L). Here, C denotes the anthocyanin content (mg/100g); A = A_1_ − A_4.5_, where A_1_ and A_4.5_ represent the absorbance values under pH 1.0 and pH 4.5 conditions, respectively; M represents the molar mass of anthocyanins (the molar mass of cyanidin-3-glucoside is 449.2 g/mol); DF indicates the dilution factor; ε refers to the molar absorptivity (the molar absorptivity of cyanidin-3-glucoside is 26,900 L/(mol·cm)); and L denotes the cuvette path length (typically 1 cm).

### 4.8. Anthocyanin Metabolites Analysis of Transgenic Tobacco

A mixed solution was used as a QC sample. During the instrumental analysis process, a QC sample is typically included every 10 analytical samples. The stability of the instrument during the project’s detection period was evaluated by overlaying and analyzing the Total Ion Chromatogram (TIC) obtained from the mass spectral analysis of the same QC sample (Figure S7).

The leaves of transgenic lines and WT were harvested and stored at −80 °C before anthocyanin metabolite analysis. Three biological replicates were analyzed for each sample. HPLC-grade methanol (MeOH) was purchased from Merck (Darmstadt, Germany). MilliQ water (Millipore, Bradford, PA, USA) was used in all experiments. All of the standards were purchased from isoReag (Shanghai, China). Formic acid was purchased from Sigma-Aldrich (St Louis, MO, USA). Hydrochloric acid was bought from Xinyang ChemicalReagent (Xinyang, China). The stock solutions of standards were prepared at the concentration of 1 mg/mL in 50% MeOH. All stock solutions were stored at −20 °C. The stock solutions were diluted with 50% MeOH to working solutions before analysis.

The sample was freeze-dried, ground into powder (30 Hz, 1.5 min). A total of 50 mg of powder was weighed and extracted with 0.5 mL methanol/water/hydrochloric acid (500:500:1, *v*/*v*/*v*). Then, the extract was vortexed for 10 min and centrifuged at 12,000× *g* at 4 °C for 3 min. The residue was re-extracted by repeating the above steps again under the same conditions. The supernatants were collected and filtered through a membrane filter (0.22 μm, Anpel) before LC-MS/MS analysis.

The sample extracts were analyzed using an UPLC-ESI-MS/MS system (UPLC, ExionLC™ AD, SCIEX, USA; MS, Applied Biosystems 6500 Triple Quadrupole, SCIEX, USA). The analytical conditions were as follows. UPLC: column, Waters ACQUITY BEH C18 (Waters, USA) (1.7 µm, 2.1 mm × 100 mm); solvent system, water (0.5% formic acid): methanol (0.5% formic acid); gradient program, 95:5 *v*/*v* at 0 min, 50:50 *v*/*v* at 6 min, 5:95 *v*/*v* at 12 min, hold for 2 min, 95:5 *v*/*v* at 14 min; hold for 2 min; flow rate, 0.35 mL/min; temperature, 40 °C; injection volume, 2 μL.

Linear ion trap (LIT) and triple quadrupole (QQQ) scans were acquired on a triple quadrupole-linear ion trap mass spectrometer (QTRAP), QTRAP^®^ 6500 + LC-MS/MS System (USA), equipped with an ESI Turbo Ion-Spray interface, operating in positive ion mode and controlled by Analyst 1.6.3 software (Sciex). The ESI source operation parameters were as follows: ion source, ESI+; source temperature, 550 °C; ion spray voltage (IS), 5500 V; and curtain gas (CUR), 35 psi, respectively. Anthocyanins were analyzed using scheduled multiple reaction monitoring (MRM). Data acquisition was performed using Analyst 1.6.3 software (Sciex). Multiquant 3.0.3 software (Sciex) was used to quantify all metabolites. Mass spectrometer parameters, including the declustering potentials (DPs) and collision energies (CEs) for individual MRM transitions were done with further DP and CE optimization. A specific set of MRM transitions was monitored for each period according to the metabolites eluted within this period.

The MWDB (Metware Database) was constructed using reference standards to perform qualitative analysis of mass spectrometry detection data. The integral peak areas of all detected samples were substituted into the linear equation of the standard curve for calculation; after further substitution into the calculation formula, the final content data of the target substance in the actual sample was obtained. Metabolite concentration in the sample (μg/g) = c×V/1,000,000/m. The variables in the formula are defined as follows: c represents the concentration value obtained by substituting the integral peak areas from the sample into the standard curve (ng/mL); V represents the volume of the solution used for extraction (μL); m represents the mass of the weighed sample (g).

Anthocyanin metabolites were detected by MetWare (http://www.metware.cn/) based on the AB Sciex QTRAP 6500 LC-MS/MS platform.

### 4.9. Statistical Analysis

All data are presented as means ± SD based on at least three biological replicates and three technical replicates. For Figure 2, significant variations (*p* < 0.05) were determined using one-way ANOVA followed by LSD post hoc tests in SPSS 18.0, assuming homogeneity of variance. For Figure 3, Figure 4 and Figure 5, significant differences (*p* < 0.05) were assessed using unpaired, two-tailed Student’s *t*-tests in Excel.

## 5. Conclusions

In this study, we identified *SoMYB1*, an R2R3-MYB transcription factor involved in the anthocyanin biosynthetic pathway of *S. oblata.* We demonstrated that the overexpression of *PJAM1502*-*SoMYB1* could induce anthocyanin biosynthesis in roots, stems, leaves, flowers, capsules and seeds of tobacco plants. Quantitative analysis revealed a significant increase in total anthocyanin content, particularly cyanidin, in transgenic tobacco leaves. These findings indicate that *SoMYB1* may serve as a target for molecular breeding applications.

## Data Availability

All data supporting the findings of this study are available within the paper and its Supplementary Information.

## Supplementary Materials

The following supporting information can be downloaded at: https://www.mdpi.com/article/10.3390/plants15162492/s1, Figure S1: The CDS sequence and the deduced amino acid sequence of *SoMYB1.* The start codon (ATG) and the stop codon (TGA) were underlined; Figure S2: The electrophoretic gel photograph of *SoMYB1* CDS (705bp) sequence amplification. 1-6 indicate positive amplification, M represents the D2000 Marker; Figure S3: The Sequencing chromatogram of the *SoMYB1* CDS region linked to the *pDONR207* vector; Figure S4: The electrophoretic gel photograph of the constructed *SoMYB1* expression vector. M: D2000; 1. BP-positive bacterial strain; 2. LR-positive bacterial strain; 3. BP-positive bacterial plasmid; 4. LR-positive bacterial plasmid; Figure S5: PCR identification of transgenic tobacco lines overexpressing *SoMYB1*. M: D2000; positions 1-7 indicate positive amplification; positions 8-9 represent positive controls; positions 10-12 indicate negative controls; Figure S6: Relative transcript levels of the *SoMYB1* gene in the leaves of WT and transgenic lines (*SoMYB1*-1, *SoMYB1*-2, *SoMYB1*-3). Data are means ± SD of at least three biological replicates, the transgenic lines and WT were considered significant at *p* < 0.05 analyzed by Student’s t-test; Figure S7: The TIC overlaps by QC sample Mass Spectrometry. The pictures of mixed solution shows that the curves of total ion current detected by metabolite were highly overlapping. The retention time and peak intensity were consistent, indicating that the signal stability number of the same sample is detected by mass spectrometry at different time. The high stability of the instrument provides an important guarantee for the repeatability and reliability of the data; Table S1: Primers in this study; Table S2: The differently-accumulation chemical compounds in leaves of transgenic lines and WT; Table S3: The results of insertion site.
