# Supplementary material for: Heterologous Expression of SoMYB1 Derived from Syringa oblata Enhances Cyanidin Biosynthesis in Tobacco
Source: Plants (Basel). 2026 Aug 17;15(16):2492. doi: 10.3390/plants15162492 (PMC13516756; doi:10.3390/plants15162492)
Supplement: Supplementary file 1 [file plants-15-02492-s001.zip › Figure S1-S7.pdf]

```
1    ATGTTAAGTACACAAGTTGAATTGAGAAAAGGTGCATGGACTGAAGAAGAAGATAAACTT  60
1    M L S T Q V E L R K G A W T E E E D K L  20
61   CTCCGGAAGTGCATTAACAAGTATGGAGAAGGAAAATGGCATCTAGTTCCTCTCAGAGCA  120
21   L R K C I N K Y G E G K W H L V P L R A  40
121  GGGTTGAACAGATGCAGGAAGAGCTGTAGGCTGAGGTGGTTAAACTATCTCAGGCCAAAT  180
41   G L N R C R K S C R L R W L N Y L R P N  60
181  ATCAAAAGAGGAGACTTTGCTCCGGATGAAGATGATCTCATTATAAGGCTTCATAACTTG  240
61   I K R G D F A P D E D D L I I R L H N L  80
241  TTAGGAAACAGATGGTCACTGATATCCGGTAGAATTCCTGGAAGAACCGGCAACGACGTG  300
81   L G N R W S L I S G R I P G R T G N D V  100
301  AAAAACCATTGGAACACCCATCTGCAGAAGAAGGTATTGGGTCGAGAAGAGGGGAAGACG  360
101  K N H W N T H L Q K K V L G R E E G K T  120
361  AAAGCCCAGAAAACCACCAAAACGACAATCTTGAGACCTCGACCTCGGACCTTCAAAAGA  420
121  K A Q K T T K T T I L R P R P R T F K R  140
421  ATTATTAATGGATCTTTTTCGTCATTAGATCAGAAAATAAATAATATTTCAGCTCTAACT  480
141  I I N G S F S S L D Q K I N N I S A L T  160
481  GATCAAAGTTCAGACATTCCATCTCCATCATCGTCAAAACAAGTAGACGATGCATGCACC  540
161  D Q S S D I P S P S S S K Q V D D A C T  180
541  CAGACGCAGTGGTGGAGTAACTTGCTCGATTCTGTCGAAATTGATGGAGAAACTGAGCCA  600
181  Q T Q W W S N L L D S V E I D G E T E P  200
601  GGATCTAAAGGAATATCTCTAGAATTGCAACAAGATGATGGCTGGAATGACTTTTCTCTT  660
201  G S K G I S L E L Q Q D D G W N D F S L  220
661  GATAAGGGCATTTGGGAATTCCTGAGTTCTGAAAATATCATATGA  705
221  D K G I W E F L S S E N I I *  234
```

Figure S1 The CDS sequence and the deduced amino acid sequence of *SoMYB1*. The start codon (ATG) and the stop codon (TGA) were underlined.

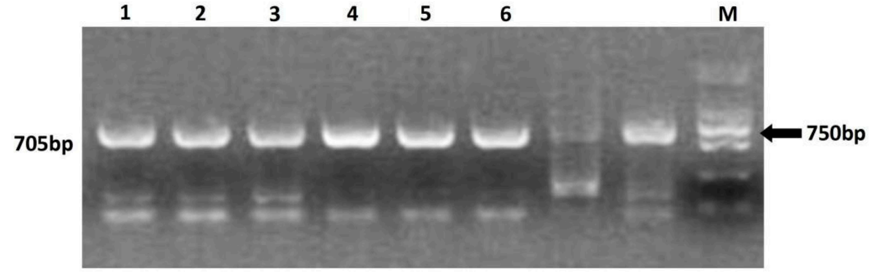

Figure S2 The electrophoretic gel photograph of *SoMYB1* CDS (705bp) sequence amplification. 1-6 indicate positive amplification, M represents the D2000 Marker.

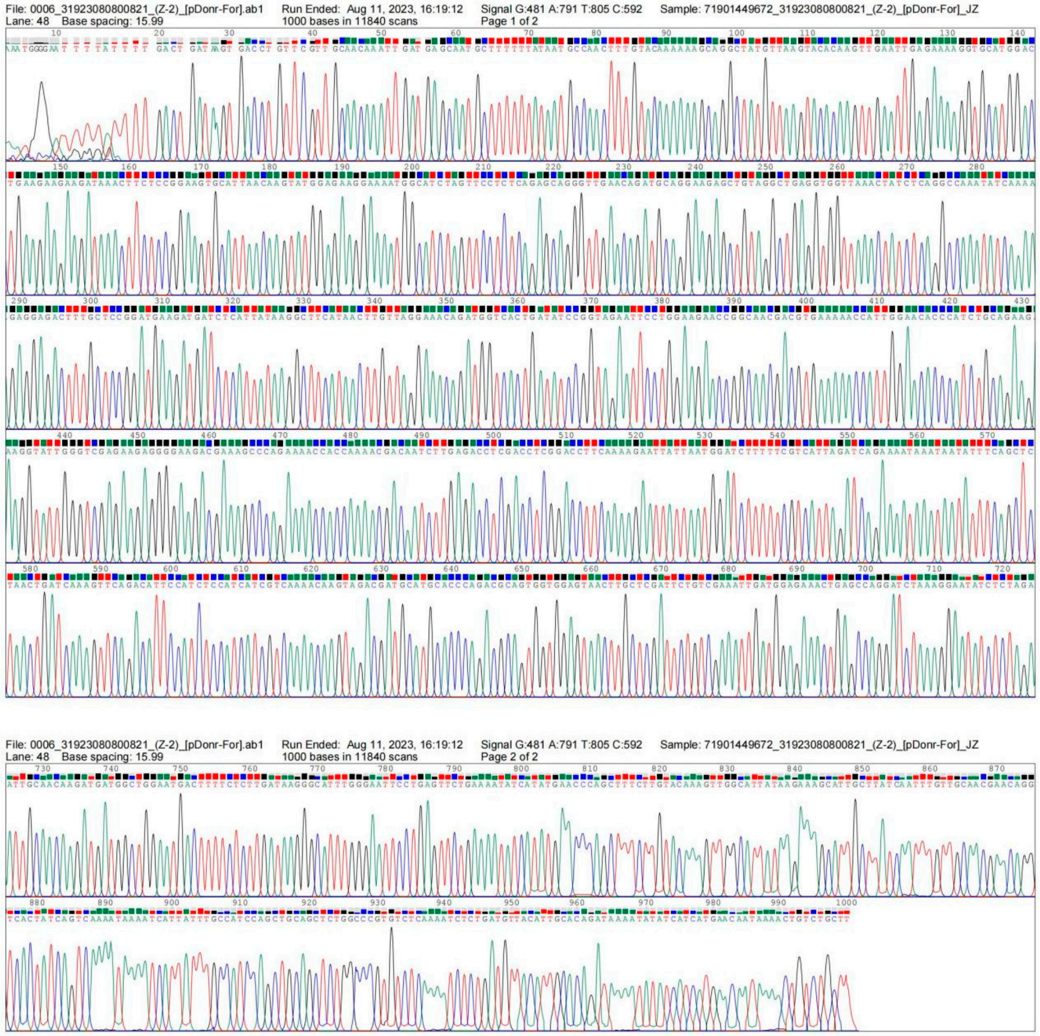

Figure S3 The Sequencing chromatogram of the *SoMYB1* CDS region linked to the *pDONR207* vector

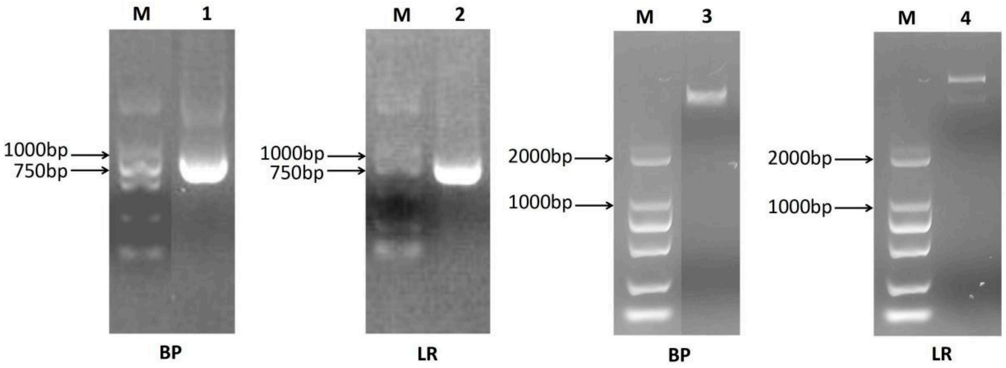

Figure S4 The electrophoretic gel photograph of the constructed *SoMYB1* expression vector. M: D2000; 1. BP-positive bacterial strain; 2. LR-positive bacterial strain; 3. BP-positive bacterial plasmid; 4. LR-positive bacterial plasmid.

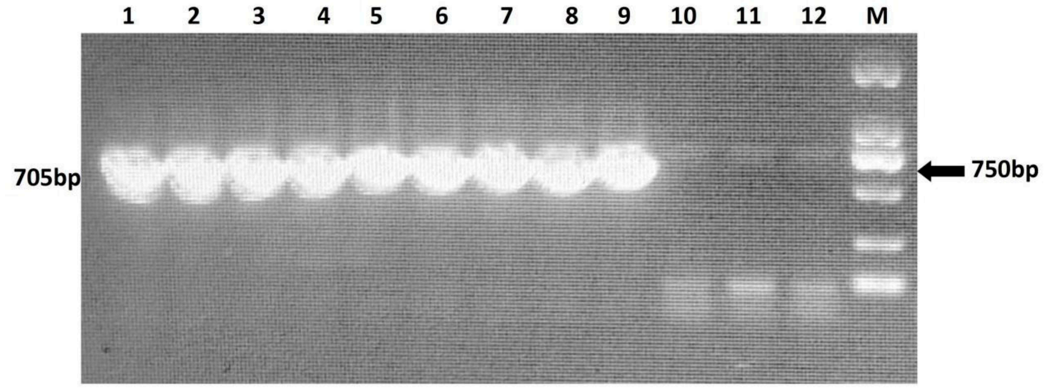

Figure S5 PCR identification of transgenic tobacco lines overexpressing *SoMYB1*. M: D2000; positions 1-7 indicate positive amplification; positions 8-9 represent positive controls; positions 10-12 indicate negative controls.

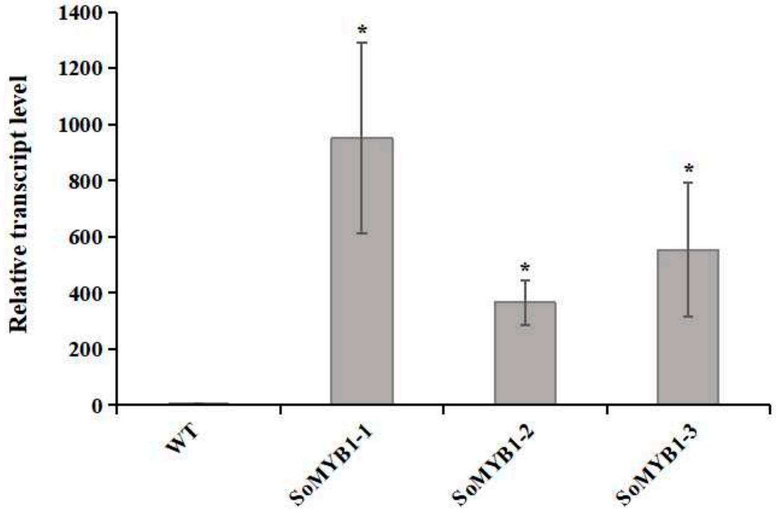

Figure S6 Relative transcript levels of the *SoMYB1* gene in the leaves of WT and transgenic lines (SoMYB1-1, SoMYB1-2, SoMYB1-3). Data are means ± SD of at least three biological replicates, the transgenic lines and WT were considered significant at $P < 0.05$ analyzed by Student's t-test.

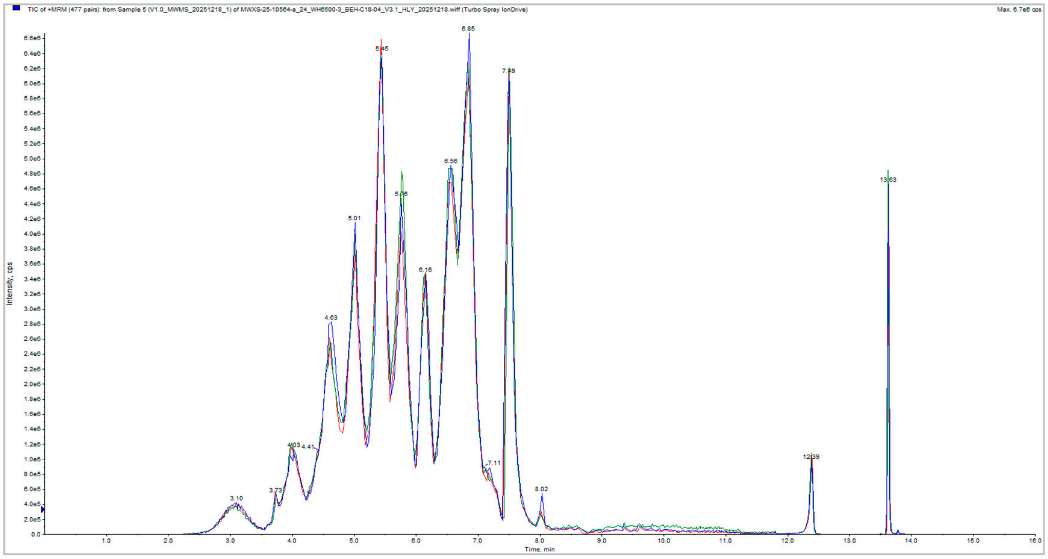

Figure S7 The TIC overlaps by QC sample Mass Spectrometry. The pictures of mixed solution shows that the curves of total ion current detected by metabolite were highly overlapping. The retention time and peak intensity were consistent, indicating that the signal stability number of the same sample is detected by mass spectrometry at different time. The high stability of the instrument provides an important guarantee for the repeatability and reliability of the data.
