# Supplementary material for: Heterologous Expression of SoMYB1 Derived from Syringa oblata Enhances Cyanidin Biosynthesis in Tobacco
Source: Plants (Basel). 2026 Aug 17;15(16):2492. doi: 10.3390/plants15162492 (PMC13516756; doi:10.3390/plants15162492)
Supplement: Supplementary file 1 [file plants-15-02492-s001.zip › Table S1.pdf]

Table S1 Primers in this study

Gene name	Primer forward	Primer reverse
SoMYB1	CTACAGATTTTCACGTGCTTATTGG	TGCATGTCTGATGTGTCTATGTCT
SoMYB1-attB	GGGGACAAGTTTGTACAAAAAAGCAG	GGGGACCACTTTGTACAAGAAAGC
	GCTATGTTAAGTACACAAGTTGA	TGGGTTCATATGATATTTTCAGAAC
qPCR primers		
qSoMYB1	CGAAAGCCCAGAAAACCACC	GTTACTCCACCACTGCGTCT
SoActin	TGGAATGTGCTGAGAGATGC	TGCTGACCGTATGAGCAAAG
qNtAN2	GAAGAAAGGTGCATGGACTG	TCTGCAGCTCTTTCTGCATC
qNtAN1b	CTTGAACACTTCTCAAACCGA	TGCTAGGGCACAATGTGAAG
qNtCHS	TTGTTCGAGCTTGTCTCTGC	AGCCCAGGAACATCTTTGAG
qNtCHI	GTCAGGCCATTGAAAAGCTC	CTAATCGTCAATGCCCCAAC
qNtF3H	CAAGGCATGTGTGGATATGG	TGTGTCGTTTCAGTCCAAGG
qNtF3′H	AGGCTCAACACTTCTCGT	CATCAACTTTGGGCTTCT
qNtF3′5′H	CGCACTACCATACTTAGGAGCCAT	CAGCATCAGGAGTAGAAGCAACAG
qNtDFR	AACCAACAGTCAGGGGAATG	TTGGACATCGACAGTTCCAG
qNtANS	TGGCGTTGAAGCTCATACTG	GGAATTAGGCACACACTTTGC
qNtUFGT	GAGTGCATTGGATGCCTTTT	CCAGCTCCATTAGGTCCTTG
qNtELF	TGAACCATCCACGACAGATTGG	TGGGCTCCTTCTCAATCTCCTT
qNt3RT	GGAGCAAACTGGGCTACCTT	CTGTTGTTGCACCCAACCTG
